# A Randomized Controlled Trial of Angiotensin-Converting Enzyme Inhibition for Skeletal Muscle Dysfunction in COPD

**DOI:** 10.1378/chest.13-2483

**Published:** 2014-02-20

**Authors:** Dinesh Shrikrishna, Rebecca J. Tanner, Jen Y. Lee, Amanda Natanek, Amy Lewis, Patrick B. Murphy, Nicholas Hart, John Moxham, Hugh E. Montgomery, Paul R. Kemp, Michael I. Polkey, Nicholas S. Hopkinson

**Affiliations:** From the National Heart & Lung Institute (NHLI) (Drs Shrikrishna, Natanek, and Hopkinson; Ms Tanner; and Prof Polkey), NIHR Respiratory Biomedical Research Unit, Royal Brompton & Harefield NHS Foundation Trust and Imperial College London; Molecular Medicine Section (Dr Shrikrishna, Lee, Natanek, Lewis, and Kemp), National Heart & Lung Institute (NHLI), Imperial College London; Guy’s and St. Thomas’ NHS Foundation Trust and NIHR Comprehensive Biomedical Research Centre (Drs Murphy and Hart) and Department of Asthma, Allergy & Respiratory Science (Prof Moxham), Division of Asthma, Allergy and Lung Biology, King’s College London; and Institute for Human Health and Performance (Prof Montgomery), University College London, London, England.

## Abstract

**BACKGROUND:**

Skeletal muscle impairment is a recognized complication of COPD, predicting mortality in severe disease. Increasing evidence implicates the renin-angiotensin system in control of muscle phenotype. We hypothesized that angiotensin-converting enzyme (ACE) inhibition would improve quadriceps function and exercise performance in COPD.

**METHODS:**

This double-blind, randomized placebo-controlled trial investigated the effect of the ACE inhibitor, fosinopril, on quadriceps function in patients with COPD with quadriceps weakness. Primary outcomes were change in quadriceps endurance and atrophy signaling at 3 months. Quadriceps maximum voluntary contraction (QMVC), mid-thigh CT scan of the cross-sectional area (MTCSA), and incremental shuttle walk distance (ISWD) were secondary outcomes.

**RESULTS:**

Eighty patients were enrolled (mean [SD], 65 [8] years, FEV_1_ 43% [21%] predicted, 53% men). Sixty-seven patients (31 fosinopril, 36 placebo) completed the trial. The treatment group demonstrated a significant reduction in systolic BP (Δ−10.5 mm Hg; 95% CI, −19.9 to −1.1; *P* = .03) and serum ACE activity (Δ−20.4 IU/L; 95% CI, −31.0 to −9.8; *P* < .001) compared with placebo. No significant between-group differences were observed in the primary end points of quadriceps endurance half-time (Δ0.5 s; 95% CI, −13.3-14.3; *P* = .94) or atrogin-1 messenger RNA expression (Δ−0.03 arbitrary units; 95% CI, −0.32-0.26; *P* = .84). QMVC improved in both groups (fosinopril: Δ1.1 kg; 95% CI, 0.03-2.2; *P* = .045 vs placebo: Δ3.6 kg; 95% CI, 2.1-5.0; *P* < .0001) with a greater increase in the placebo arm (between-group, *P* = .009). No change was shown in the MTCSA (*P* = .09) or ISWD (*P* = .51).

**CONCLUSIONS:**

This randomized controlled trial found that ACE inhibition, using fosinopril for 3 months, did not improve quadriceps function or exercise performance in patients with COPD with quadriceps weakness.

**TRIAL REGISTRY:**

Current Controlled Trials; No.: ISRCTN05581879; URL: www.controlled-trials.com

Skeletal muscle impairment is a key extrapulmonary complication of COPD, affecting approximately one-third of patients independent of the degree of airflow obstruction.^1,2^ In particular, quadriceps weakness in COPD has been associated with reduced exercise capacity,^3^ impaired quality of life,^4^ and mortality in patients with moderate to severe disease.^5^ Importantly, pulmonary rehabilitation, which improves exercise performance and reduces health-care utilization, also increases quadriceps strength.^6,7^

The mechanisms responsible for skeletal muscle dysfunction in COPD remain a matter of controversy and are likely multifactorial, but there is evidence that chronic activation of the IM renin-angiotensin system may be a key pathophysiologic pathway.^8^ In animal models,^9‐11^ angiotensin II promotes muscle loss via an inhibitory effect on the insulin-like growth factor (IGF)-1 system and stimulation of a catabolic pathway mediated by two ubiquitin ligases, the atrogenes: muscle RING finger protein-1 and atrogin-1. Through ubiquitin-proteasome degradation, these ligases have been postulated to play a key role in the muscle atrophy observed in patients with COPD.^12^

In addition, an endogenous reduction in serum and tissue angiotensin-converting enzyme (ACE) levels as a result of polymorphism of the human ACE gene has been associated with an enhanced endurance phenotype^13^ with the presence of a deletion allele (D) shown to correlate with greater quadriceps strength in COPD.^14^ A polymorphism determining a reduction in bradykinin receptor expression has also been associated with a reduced fat-free mass and quadriceps strength in patients with COPD.^15^

Further evidence has come from observational studies in hypertensive cohorts where treatment with an ACE inhibitor has been associated with increased locomotor muscle size^16^ and strength.^17^ This is supported by randomized controlled trials where ACE inhibition has increased 6-min walking distance in elderly subjects^18^ and angiotensin II receptor blockade has shown a trend toward an improvement in quadriceps strength in subjects with COPD.^19^

Given this evidence base and the increasing focus toward the development of pharmacotherapy targeting skeletal muscle,^20,21^ we hypothesized that ACE inhibition would have a beneficial effect on quadriceps function in patients with COPD. To strengthen the design of the trial we used a stratified medicine approach, selecting patients with quadriceps weakness using the cutoff that has been found to be associated with increased mortality in COPD.^5^ Quadriceps endurance, measured by repetitive magnetic stimulation, was used as the primary outcome due to the association of reduced ACE levels with greater endurance in healthy subjects^13^ and the advantages of a nonvolitional test in the context of a population with severe airflow limitation.^22^

## Materials and Methods

### Participants

The study was approved by the Joint University College London Committees on the Ethics of Human Research (reference number 08/H0715/90), and each participant gave informed written consent. Study inclusion criteria were patients diagnosed with COPD based on GOLD (Global Initiative for Chronic Obstructive Lung Disease) criteria^23^ and the presence of quadriceps weakness defined as a quadriceps maximum voluntary contraction (QMVC) in kilograms < 120% of the patient’s BMI.^5^ Exclusion criteria were patients within 3 months of pulmonary rehabilitation or 1 month of an exacerbation, and those with a comorbidity including cardiac failure, diabetes, renal disease, or rheumatoid arthritis. Patients receiving ACE inhibitors, angiotensin II receptor blockers, or warfarin (because the study entailed a vastus lateralis biopsy) were also excluded.

### Study Design

The study was a double-blind, randomized, placebo-controlled, parallel-group trial. Patients were randomly allocated to either ACE inhibitor (fosinopril 10 mg, once a day) or placebo (lactose) for a 3-month period. Resting BP and renal function were reviewed at 1 week by an independent assessor and if satisfactory, the daily dose was increased to two capsules (fosinopril 20 mg maximum or placebo). Dose was not escalated if the systolic BP was < 110 mm Hg. A pharmacy controlled, 1:1 randomization in blocks of four using consecutive numbers was performed by the Clinical Trials Department, Royal Free Hampstead NHS Trust UK. Primary outcomes were change in quadriceps endurance measured nonvolitionally by repetitive magnetic stimulation^22^ and vastus lateralis atrophy signaling using atrogene expression from biopsies taken at baseline and 3 months. Secondary outcomes included change in QMVC, quadriceps twitch force (TwQ), mid-thigh CT scan of the cross-sectional area (MTCSA), incremental shuttle walk distance (ISWD), health status, and serum inflammatory markers. Measurements of fat-free mass, BP, lung function, and anthropometrics were also made and a single assessment of baseline physical activity was conducted using a multisensor armband worn for 1 week as previously described.^2^ Further details of the trial protocol and methods are available online (e-Appendix 1, e-Tables 1, 2).

### Analysis and Statistics

Detecting a 25% increase in time to fatigue in the fosinopril vs placebo groups, with an 80% power at the 5% significance level, would require 54 patients randomized on a 1:1 basis. To allow for a 30% dropout rate, 80 patients were targeted for recruitment. Data were normally distributed and a per-protocol analysis was conducted using paired or independent *t* tests. Response variables were tested by analysis of variance with post hoc correction for more than two groups. Multiple linear regression analysis was performed to identify baseline parameters influencing response to treatment. Analysis was performed using StatView 5.0 (Abacus Concepts) with *P* < .05 considered statistically significant.

## Results

**Figure 1 –:**
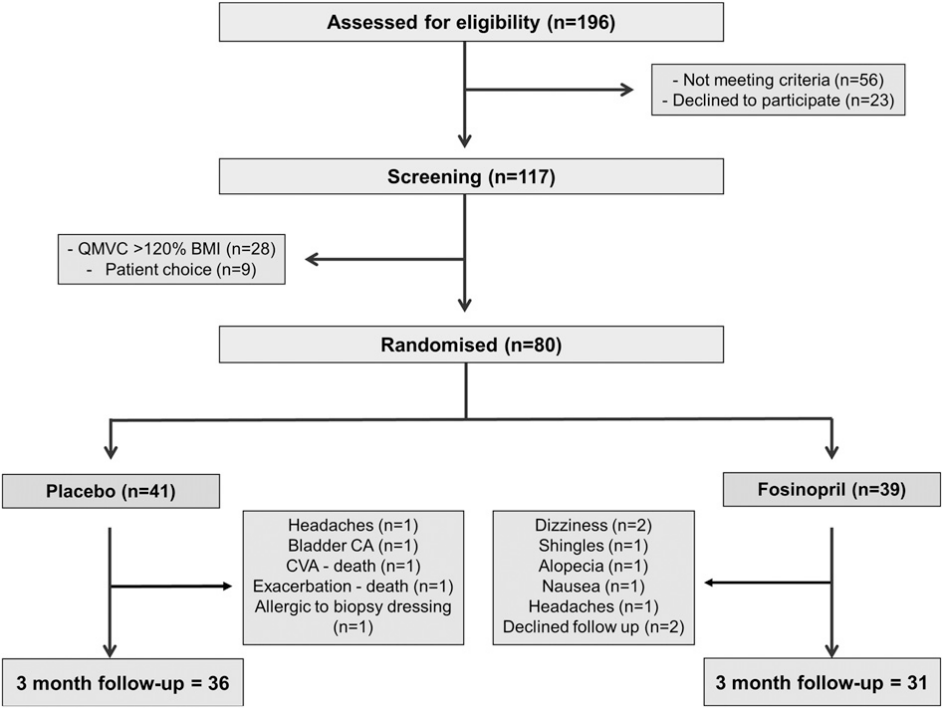
*CONSORT diagram for enrolment and follow-up. CA = carcinoma; CVA = cerebrovascular accident; QMVC = quadriceps maximum voluntary contraction*.

One hundred seventeen patients were screened for study participation, and 80 patients underwent randomization. There were eight withdrawals from the treatment group and five from the placebo group. Further details are shown in the CONSORT diagram (Fig 1).

### Baseline Anthropometrics and Muscle Measurements

The placebo and treatment groups were well matched for age, sex, and lung function parameters and there were no statistically significant baseline differences in body composition and quadriceps muscle function (Table 1). There were also no significant baseline differences in vastus lateralis atrogene expression and serum measurements between the groups (e-Table 3).

**TABLE 1 ]:** Demographic and Baseline Clinical Characteristics of Placebo and Treatment Groups

	Group, Mean (SD)		
Clinical Characteristics	Placebo (n = 41)	Treatment (n = 39)	*P* Value
Age, y	64.6 (7.3)	66.3 (8.2)	.33
Sex, male (female)	23 (18)	19 (20)	.51
BMI, kg/m^2^	24.3 (4.0)	25.0 (5.8)	.51
FFMI, kg/m^2^	17.0 (2.1)	17.3 (2.6)	.60
Smoking, pack-y	53.3 (25.1)	49.8 (33.1)	.59
Current smokers, %	24	28	.70
Long-acting β agonist, %	93	82	.15
Long-acting anticholinergic, %	88	87	.93
Inhaled corticosteroid, %	90	82	.15
Oral corticosteroid, ≥ 5 mg/d, %	2	5	.53
FEV_1_ % predicted	40.1 (20.6)	45.8 (20.5)	.22
Dlco % predicted	41.8 (20.9)	44.0 (19.2)	.64
RV to TLC ratio, %	58.2 (9.9)	55.8 (10.7)	.29
Pao_2_, kPa	9.7 (1.4)	9.6 (1.4)	.82
Paco_2_, kPa	5.2 (0.6)	5.1 (0.4)	.18
SGRQ, symptoms	56.5 (23.7)	49.6 (21.3)	.17
SGRQ, activity	70.8 (25.8)	71.1 (17.2)	.96
SGRQ, impacts	40.8 (22.8)	31.3 (16.2)	.04
SGRQ, total	52.5 (22.0)	46.4 (14.9)	.15
CAT score	22.8 (8.5)	20.8 (8.1)	.34
Daily step count	4499 (3462)	4504 (3109)	.99
PAL	1.4 (0.18)	1.4 (0.16)	.66
Systolic BP, mm Hg	134 (15)	138 (19)	.35
Diastolic BP, mm Hg	85 (10)	85 (11)	.84
β-Blocker, %	2	0	.33
Calcium channel blocker, %	7	10	.65
Diuretic, %	0	2	.31
Serum NT-proBNP, ng/L	109.0 (99.8)	105.0 (64.0)	.85
ACE genotype: DD, ID, II, %	39,44,17	38,46,16	.94
QMVC, kg	24.9 (4.9)	25.0 (7.4)	.98
TwQ, kg	10.7 (3.0)	9.7 (3.4)	.21
MTCSA, cm^2^	93.3 (22.4)	93.0 (26.1)	.96
Endurance half-time, s	61.2 (35.5)	70.6 (31.9)	.30
ISWD, m	247 (132)	242 (128)	.87

ACE = angiotensin-converting enzyme; CAT = COPD assessment test; Dlco = diffusing capacity of the lung for carbon monoxide; FFMI = fat free mass index; ISWD = incremental shuttle walk distance; MTCSA = mid-thigh CT scan of the cross-sectional area; NT-proBNP = N-terminal pro-B-type natriuretic peptide; PAL = physical activity level; QMVC = quadriceps maximum voluntary contraction; RV = residual volume; SGRQ = St. George’s Respiratory Questionnaire; TLC = total lung capacity; TwQ = quadriceps twitch force.

### Physiologic Outcomes Following ACE Inhibition

#### Quadriceps Function:

**Figure 2 –:**
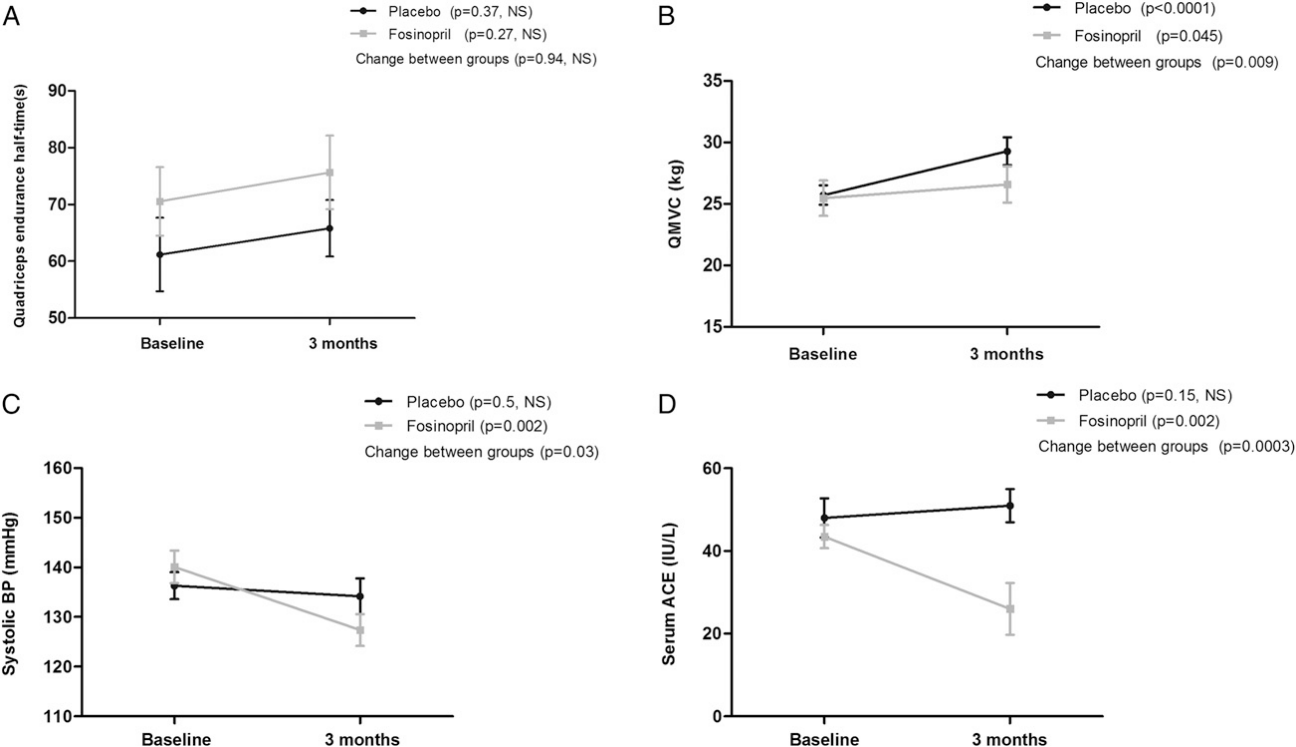
*A, Quadriceps endurance following 3 mo ACE inhibition vs placebo (data shown as mean with cross bars representing the SEM). B, QMVC following 3 mo ACE inhibition vs placebo (data shown as mean with cross bars representing SEM). C, Systolic BP following 3 mo ACE inhibition vs placebo (data shown as mean with cross bars representing SEM). D, Serum ACE activity following 3 mo ACE inhibition vs placebo (data shown as mean with cross bars representing SEM). ACE = angiotensin-converting enzyme; NS = not significant. See Figure 1 legend for expansion of other abbreviations*.

At 3 months, there was no significant difference in quadriceps endurance assessed by repetitive magnetic stimulation (fosinopril: Δ5.1 s; 95% CI, −4.3-14.5; *P* = .27 vs placebo: Δ4.6 s; 95% CI, −5.8-15.1; *P* = .37 [between-group difference, 0.5 s; 95% CI, −13.3-14.3; *P* = .94]) (Fig 2A). QMVC improved in both groups (fosinopril: Δ1.1 kg; 95% CI, 0.03-2.2; *P* = .045 vs placebo: Δ3.6 kg; 95% CI, 2.1-5.0; *P* < .0001) with a greater increase in the placebo arm (between-group difference, 2.5 kg; 95% CI, 0.7-4.3; *P* = .009) (Fig 2B). TwQ response did not differ in the placebo vs treatment group (fosinopril: Δ−0.28 kg; 95% CI, −1.0-0.45; *P* = .43 vs placebo: Δ0.57 kg; 95% CI, 0.01-1.1; *P* = .046 [between-group difference, 0.85 kg; 95% CI, −1.7-0.03; *P* = .06]). MTCSA also showed no significant differences at 3 months (fosinopril: Δ−0.60 cm^2^; 95% CI, −2.1-0.91; *P* = .42 vs placebo: Δ1.0 cm^2^; 95% CI, −0.21-2.2; *P* = .10 [between-group difference, −1.6 cm^2^; 95% CI, −3.5-0.27; *P* = .09]).

#### Exercise Capacity, BP, and Lung Function:

There was no significant change in ISWD at 3 months in the two groups (fosinopril: Δ7.1 m; 95% CI, −5.5-19.7; *P* = .26 vs placebo: Δ17.1 m; 95% CI, −10.6-44.8; *P* = .22 [between-group difference, −10 m; 95% CI, −39.8-19.8; *P* = .51]). A significant reduction was demonstrated in systolic BP in the treatment arm compared with placebo (fosinopril: Δ−12.7 mm Hg; 95% CI, −20.3 to −5.1; *P* = .002 vs placebo: Δ−2.2 mm Hg; 95% CI, −8.1-3.7; *P* = .46 [between-group difference, −10.5 mm Hg; 95% CI, −19.9 to −1.1; *P* = .03]) (Fig 2C). Diastolic BP was also reduced in the treatment group (fosinopril: Δ−7.0 mm Hg; 95% CI, −11.5 to −2.4; *P* = .004 vs placebo: Δ−1.2 mm Hg; 95% CI, −5.2-2.9; *P* = .56 [between-group difference, −5.8 mm Hg; 95% CI, −11.7-0.11; *P* = .05]). Lung function parameters including FEV_1_ % predicted, diffusing capacity of the lung for carbon monoxide % predicted, residual volume-to-total lung capacity ratio, and arterial blood gas measurements showed no significant change between groups at 3 months, as shown in Table 2. Health-related quality-of-life measures, which included St. George’s Respiratory Questionnaire (SGRQ) and COPD assessment test (CAT) score, also did not vary significantly between groups (Table 2).

**TABLE 2 ]:** Physiologic and HRQOL Measurements Before and After 3 Mo of ACE Inhibition

	Placebo Group, mean (SEM) (n = 36)		Treatment Group, mean (SEM) (n = 31)		Change Between Groups *P* Value
Measurements	Baseline	3 Mo	Baseline	3 Mo	
TwQ, kg^a^	10.7 (0.5)	11.3 (0.6)	10.1 (0.8)	9.8 (0.8)	.06
MTCSA, cm^2^	94.1 (3.7)	95.1 (3.6)	94.5 (4.9)	93.9 (4.9)	.09
ISWD, m	247.4 (23.8)	264.5 (29.2)	241.9 (23.0)	249.0 (23.3)	.51
Diastolic BP, mm Hg	84.8 (1.6)	83.6 (1.8)	85.9 (2.1)	78.9 (2.3)	.05
FEV_1_ % predicted	40.0 (3.5)	42.7 (3.7)	44.8 (3.6)	46.1 (3.9)	.50
Dlco % predicted	43.3 (3.7)	44.5 (3.9)	44.5 (3.7)	44.2 (3.7)	.20
RV to TLC ratio, %	58.4 (1.7)	56.3 (1.8)	56.2 (1.9)	55.0 (2.0)	.35
Pao_2_, kPa	9.6 (0.2)	9.5 (0.3)	9.6 (0.3)	9.6 (0.3)	.77
Paco_2_, kPa	5.3 (0.1)	5.3 (0.1)	5.1 (0.1)	5.1 (0.1)	.79
SGRQ, symptoms	55.4 (4.0)	53.6 (4.3)	50.3 (3.7)	54.3 (4.0)	.25
SGRQ, activity	70.3 (4.4)	68.8 (4.1)	72.0 (3.1)	70.5 (4.1)	.99
SGRQ, impacts	40.2 (3.8)	39.6 (3.7)	32.2 (2.8)	34.5 (3.4)	.28
SGRQ, total	51.9 (3.7)	50.8 (3.6)	47.3 (2.6)	48.8 (3.1)	.25
CAT score	22.2 (1.5)	19.7 (1.6)	21.0 (1.6)	20.7 (1.5)	.18

HRQOL = health-related quality of life. See Table 1 legend for expansion of other abbreviations.

a TwQ: placebo (n = 32), treatment (n = 25) due to below supramaximal twitch response.

### Molecular Outcomes Following ACE Inhibition

#### Vastus Lateralis messenger RNA Expression:

At 3 months, no significant differences were observed in vastus lateralis atrogin-1 messenger RNA (mRNA) expression (fosinopril: Δ−0.18 arbitrary units [AU]; 95% CI, −0.41-0.04; *P* = .11 vs placebo: Δ−0.15 AU; 95% CI, −0.35-0.04; *P* = .12 [between-group difference, −0.03 AU; 95% CI, −0.32-0.26; *P* = .84]) or muscle RING finger protein-1 mRNA expression (fosinopril: Δ0.09 AU; 95% CI, −0.21-0.39, *P* = .55 vs placebo: Δ−0.13 AU; 95% CI, −0.32-0.05; *P* = .14 [between-group difference, 0.22 AU; 95% CI, −0.11-0.55; *P* = .18]). Vastus lateralis IGF-1 mRNA expression also showed no significant difference between groups (0.04 AU; 95% CI, −0.38-0.46; *P* = .84) at 3 months. Further data showing no significant difference in vastus lateralis transforming growth factor-β, MyoD, MHC I, IIA, and IIX mRNA expression and protein levels of phosphorylated 4EBP-1 are shown in the online supplement (e-Table 4).

#### Serum Measurements:

The treatment group demonstrated a significant reduction in serum ACE activity compared with placebo (fosinopril: Δ−17.4 IU/L; 95% CI, −28.1 to −6.8; *P* = .002 vs placebo: Δ3.0 IU/L; 95% CI, −1.2-7.1; *P* = .15 [between-group difference, −20.4 IU/L; 95% CI, −31.0 to −9.8; *P* = .0003) (Fig 2D). No significant differences were found in serum IGF-1, high-sensitivity C-reactive protein, N-terminal pro-B-type natriuretic peptide, fibrinogen, or serum inflammatory cytokines between the groups as shown in e-Table 4. A post hoc stratification based on ACE genotype did not influence response to treatment as detailed online (e-Tables 5, 6). Multiple linear regression analysis did not identify any baseline demographic, anthropometric, lung function, or physical activity variables influencing response to treatment. Data on dropouts from the study are shown in e-Table 7.

## Discussion

ACE inhibition by fosinopril did not have a beneficial effect on quadriceps muscle function over a 3-month period in patients with COPD selected for quadriceps weakness, although measurements of BP and serum ACE activity confirmed both adherence and biologic activity of the drug. Moreover, we were unable to detect any changes in atrophy signaling pathways in the participants. The present data do not support the use of ACE inhibitors alone to augment muscle phenotype in patients with COPD.

These findings were unexpected given the evidence for ACE inhibition on skeletal muscle function, and reinforce the importance of conducting prospective controlled trials. An observational study by Onder et al^17^ assessed the relationship between ACE inhibitor use and muscle strength in 641 elderly hypertensive women. They found that at 3 years’ follow-up, participants taking an ACE inhibitor continuously had a lower mean decline in both knee extensor muscle strength and walking speed than those using other antihypertensives and those not on any antihypertensive medications. Intermittent use of ACE inhibitors was also associated with a significantly larger decline in walking speed compared with continuous use. The study group had poor mobility but no concomitant heart failure at baseline. In addition, cross-sectional data from 2,431 hypertensive subjects was used to evaluate whether ACE inhibitor treatment is associated with a larger lower extremity muscle mass compared with the use of other antihypertensive medications,^16^ and found that lower extremity muscle mass was larger in the ACE inhibitor group in a manner proportional to the length of use.

Interventional studies of ACE inhibition have also suggested a treatment effect. A randomized controlled trial in 95 elderly subjects with self-reported difficulties in mobility, showed that 5 months of perindopril treatment significantly improved 6-min walk distance (31.4 m; 95% CI, 10.8 m-51.9 m; *P* = .003) compared with placebo.^18^ Interestingly, this cohort included current and ex-smokers and the improvement was observed in the absence of heart failure in the participants. In addition, a small randomized controlled trial of 21 subjects with COPD evaluating the effects of 4 weeks’ treatment with enalapril on exercise performance found a significant improvement in O_2_ pulse and peak work rate in the treatment group.^24^

There are a number of possible factors that may explain why ACE inhibition was not effective in the present study given previous data. These are discussed here and include the patient population studied, the influence of physical inactivity, and the possibility that ACE inhibition created a more “benign” IM environment, effectively removing a training stimulus.

### Patient Selection

We adopted a stratified medicine approach, selecting a quadriceps weakness patient phenotype, to focus on those patients with COPD with a level of skeletal muscle dysfunction known to be associated with worse survival.^5^ It may, however, be that at this stage the weakest patients have a limited ability to respond to treatment and the low physical activity level found at baseline may reflect this. The level of inactivity could explain the discrepancy between the present data and the effect of ACE inhibitors in relatively healthy populations being treated for hypertension.^17^ There is also evidence that an exercise stimulus may be needed for ACE inhibition to promote adaptive changes, such as an increase in capillary density, in skeletal muscle.^25^ Therefore, the current data do not preclude the possibility that the use of ACE inhibition in the context of pulmonary rehabilitation may yield benefit.

Skeletal muscle impairment in COPD involves both fiber atrophy and fiber shift away from an oxidative, fatigue-resistant phenotype.^26^ In any individual, these processes occur to a varying extent with different effects on muscle function. The D allele of the ACE (I/D) polymorphism, which is associated with higher ACE activity, was associated with greater quadriceps strength in patients with COPD.^14^ The effects of ACE inhibition may therefore counteract strength adaptations seen in COPD, in favor of achieving a more aerobic phenotype. This trial was not prospectively powered for stratification by ACE genotype, so although post hoc analysis did not identify a genotype-specific influence on quadriceps function, this method of stratification would be an important consideration for future studies.

### Effect of ACE Inhibition on IM Environment

The improvements observed in quadriceps strength in the current trial warrant further discussion. As subjects were included based on the presence of quadriceps weakness, it is not completely unexpected that a potential placebo effect with regression to the mean would be observed in relation to volitional quadriceps strength, although more detailed monitoring of physical activity throughout the trial period would be needed to support this. The existence of a placebo effect has been well documented in the context of functional exercise capacity in clinical trials of ACE inhibition in heart failure^27^ and in other studies of physical performance.^28^ However, the finding that this effect was greater in the placebo arm of the trial was unexpected. While this could be a chance effect, this seems unlikely because of the high level of statistical significance shown (*P* = .009). A possible reduction in skeletal muscle blood flow secondary to a reduced systemic BP may explain the attenuated response observed with treatment. Of note, captopril has been shown to increase maximal blood lactate during exercise and reduce exercise capacity in normotensive, sedentary rats.^29^

Despite evidence from animal models of the ability to prevent angiotensin II-induced cachexia via IGF-1 overexpression and atrogene downregulation,^10^ ACE inhibition was not shown to have a translational benefit in patients with COPD. This demonstrates that, at least over a 3-month period, fosinopril does not alter atrophy signaling at a tissue level, despite a clear systemic effect on BP and serum ACE activity. Importantly, there is evidence to suggest this systemic effect would have inhibited tissue ACE.^30^ However, local tissue renin-angiotensin system can generate angiotensin II, independent of ACE activity, through serine proteases such as chymase and cathepsin G^31^ and this may explain the lack of influence of ACE inhibition on skeletal muscle. The timing of biopsies may have also influenced the ability to detect changes following ACE inhibition and, therefore, early biopsies, where feasible, may guide further mechanistic work in this area.

### Critique of the Method

A strength of this study was that the quadriceps assessment was comprehensive including both volitional and nonvolitional physiologic outcomes as well as molecular outcomes. Although no significant changes were shown in the activity domain of the SGRQ, the influence of treatment on physical activity level was not objectively assessed in this trial. The baseline inclusion of physical activity monitoring did, however, objectively confirm that both groups were well matched for their level of physical activity when commencing the trial. Importantly, this activity level was in keeping with published data on patients from other northern European countries,^32^ highlighting the overall generalizability of the study population findings. However, the inclusion of more patients with varying degrees of muscle impairment may have enabled a wider assessment of potential responders.

Finally, a 3-month study duration was chosen as this was comparable to the time period used in other relevant studies^19,24^ and would minimize potential confounding by intercurrent exacerbations. However, the time needed for skeletal muscle adaptation following pharmacotherapy in these patients is unclear, with ACE inhibitor treatment duration ranging from 10 weeks to 12 months for studies of improved 6-min walk distance in heart failure.^27^ The potential role of longer-term therapy remains to be evaluated in further trials.

## Conclusions

In summary, despite a strong theoretical basis for the study, we found that the ACE inhibitor fosinopril did not improve quadriceps function over a 3-month period, in a COPD population selected for quadriceps weakness. This study does not support a role for ACE inhibition alone in the treatment of skeletal muscle dysfunction in patients with COPD. Future work should focus on the use of pharmacotherapy during pulmonary rehabilitation to investigate augmenting exercise effects in these patients.

## Supplementary Material

Online Supplement

## ABBREVIATIONS

ACE: angiotensin-converting enzyme
AU: arbitrary unit
CAT: COPD assessment test
IGF: insulin-like growth factor
ISWD: incremental shuttle walk distance
mRNA: messenger RNA
MTCSA: mid-thigh CT scan of the cross-sectional area
QMVC: quadriceps maximum voluntary contraction
SGRQ: St. George’s Respiratory Questionnaire
TwQ: quadriceps twitch force
