# Supplementary material for: A Randomized Controlled Trial of Angiotensin-Converting Enzyme Inhibition for Skeletal Muscle Dysfunction in COPD
Source: Chest. 2014 Feb 20;146(4):932–40. doi: 10.1378/chest.13-2483 (PMC4188149; doi:10.1378/chest.13-2483)
Supplement: Supplementary file 1 [file chest_146_4_932_ds01.pdf]

CHEST Online Supplement

A Randomized Controlled Trial of Angiotensin-Converting Enzyme Inhibition for Skeletal Muscle Dysfunction in COPD

Dinesh Shrikrishna, PhD; Rebecca J. Tanner, BSc; Jen Y. Lee, PhD; Amanda Natanek, PhD; Amy Lewis, PhD; Patrick B. Murphy, PhD; Nicholas Hart, PhD; John Moxham, PhD; Hugh E. Montgomery, PhD; Paul R. Kemp, PhD; Michael I. Polkey, PhD; and Nicholas S. Hopkinson, PhD

CHEST 2014;146(4):932-940

e-Appendix 1.

METHODS

COPD patients were recruited through outpatient clinics at the Royal Brompton, King's College and St Thomas' Hospitals, and through public spirometry events conducted on World COPD and No-Smoking days. Data collection and analysis were conducted at the Royal Brompton Hospital and Imperial College London. The trial schedule is summarised in e-Table 1.

Pulmonary function testing

Patients had a baseline assessment of pulmonary function, including spirometry, plethysmographic lung volumes, carbon monoxide diffusing capacity (TLco) (CompactLab system; Jaeger, Wurzburg, Germany) and arterial blood gases, determined in accordance with American Thoracic Society (ATS)/European Respiratory Society (ERS) recommendations [1-3].

Blood pressure, anthropometrics, fat free mass measurements

Blood pressure was recorded at baseline using an automated blood pressure monitor (Omron M6, Omron Healthcare Europe, Hoofddorp, The Netherlands). The subject was rested for at least 20 minutes prior to blood pressure measurement and the average of three readings was used. Baseline anthropometrics and renal function were also recorded. Fat free mass index (FFMI), was assessed by bioelectrical impedance analysis (BodyStat QuadScan 4000; BodyStat, Douglas, United Kingdom). The bioelectrical impedance of body tissues can be used to estimate total body water, as electricity is conducted by dissolved ions. A two-compartment model was adopted which assumes that adipose tissue contains no water and that FFM is of particular percentage water. Single frequency, 50kHz, bioelectrical impedance values were incorporated into a validated disease specific regression equation including height, weight and gender to calculate fat free mass (FFM) [4]. A single assessment of baseline physical activity was also made, using a multisensor

Online supplements are not copyedited prior to posting.

© 2014 AMERICAN COLLEGE OF CHEST PHYSICIANS. Reproduction of this article is prohibited without written permission from the American College of Chest Physicians. See online for more details. **DOI:** 10.1378/chest.13-2483

biaxial accelerometer armband (SenseWear, BodyMedia; Pittsburgh, PA) worn for one week as previously described [5].

Quadriceps measurements

Quadriceps maximal voluntary contraction

Quadriceps strength was assessed by maximum voluntary contraction (QMVC) [6]. A modified chair was used with the subject's knee fixed at 90 degrees and an inextensible strap connecting the ankle of their dominant leg to a strain gauge. The strain gauge signal was amplified and viewed onscreen using CHART software (Labchart version 7.1, PowerLab Analogue-Digital Converter, AD instruments, Oxfordshire, UK). The subject performed at least 3 sustained maximal isometric quadriceps contractions (5-10 seconds duration) with a gap of approximately 30 seconds between each contraction. Vigorous encouragement was given and the QMVC was recorded as the highest tension sustained for 1 second.

Quadriceps twitch force

Magnetic femoral nerve stimulation was used to determine unpotentiated twitch quadriceps force (TwQ), as a non-volitional measure of quadriceps strength [7]. A stimulus response curve was used to confirm supramaximality and the mean of 5 stimulations at 100% stimulator output was taken.

Quadriceps endurance

Non-volitional quadriceps endurance was assessed using repetitive magnetic stimulation with a Magstim Rapid 2 stimulator and a flexible mat coil wrapped around the quadriceps [8]. Subjects received 60 trains of stimulation, at a frequency of 30Hz, 2 seconds on, 3 seconds off, with the % stimulator output determined as that able to produce 20% of the subject's QMVC at baseline. The exponential decay in force produced by consecutive trains was used to assess endurance. Subjects also performed an incremental shuttle walk test (ISWT) [9] and health-related quality of life was determined using the St. George's Respiratory Questionnaire (SGRQ) and the COPD assessment test (CAT) score [10].

Mid-thigh Computed Tomography (CT) Cross-sectional area

Mid-thigh cross-sectional area (MT_{CSA}) was measured by Computed Tomography (CT) as previously described [5]. The subject was positioned supine and a CT was performed on a 64-slice CT scanner (Siemens SOMATOM Sensation 64, Erlangen, Germany). A single section of the mid-thigh at a predefined level was obtained using the following acquisition parameters: 50mAs, 120kVp. A modified protocol was adopted to deliver a reduced radiation amount per scan. Images were viewed and CT cross-sectional areas

Online supplements are not copyedited prior to posting.

© 2014 AMERICAN COLLEGE OF CHEST PHYSICIANS. Reproduction of this article is prohibited without written permission from the American College of Chest Physicians. See online for more details. **DOI:** 10.1378/chest.13-2483

calculated using Digital Imaging Communications in Medicine viewing software (DicomWorks, version 1.3;*http://dicom.online.fr*) at standardised window settings for soft tissue visualisation (centre 40 HU, window width 380 HU).

Vastus Lateralis biopsy and RT- qPCR analysis

Muscle biopsies were taken from the *vastus lateralis* of the non-dominant leg using the Bergstrom technique [11]. Following infiltration with local anaesthetic, a 1cm incision was made in the skin and a Bergstrom needle used to obtain the muscle tissue. Samples were snap frozen in liquid nitrogen and stored at -80°C. For RNA extraction, muscle samples were suspended in trizol (Sigma, UK) and homogenised using 1.4mm ceramic beaded tubes (Stretton Scientific, UK) in a Precellys 24 homogeniser (Peqlab, Erlangen, Germany). The samples were then centrifuged, with the resulting supernatant used for extraction. RNA concentration was quantified with a spectrophotometer (Nanodrop ND1000, Wilmington, USA). First strand cDNA was synthesised using Superscript II Reverse Transcriptase (Invitrogen). Real-time quantitative PCR (RT-qPCR) was performed as previously described [12], in duplicate on each sample, testing for MuRF-1, Atrogin-1, IGF-1, MyoD, TGF-β, MHC I, MHC IIA, MHC IIX and the reference housekeeping gene, human RPLPO, using a 20ul reaction of Fast SYBR Green Quantitative PCR Kit (Applied Biosystems, UK) and the primer pair (2pmol/ul) in 96 well plates (MicroAmp, Fast optical 96 well reaction plate (0.1 ml) (Applied Biosystems, UK). RT-qPCR reactions were run using a 7500 Fast Real-time PCR System (Applied Biosystems, UK), with the following cycle program: 95 ºC for 5 minutes, then 40 cycles of 95 ºC for 10 seconds, 60°C for 30 seconds. PCR products were run on a 2% agarose gel to confirm product size. The mRNA levels for the genes analysed were normalised to human RPLPO mRNA, with the values then log transformed to obtain a normal distribution. Primer sequences are detailed below (e-Table 2).

For protein extraction, muscle samples were homogenised in lysis buffer containing protease and phosphatase inhibitors (Sigma, UK). A Bradford assay was then used to measure protein concentration against BSA standards and to determine levels of phosphorylated 4EBP-1 the protein lysate was analysed using an enzyme linked immunosorbent assay (ELISA) kit, (Invitrogen) containing a specific 4EBP-1 detection antibody. The absorbance at 450nm was measured using a Bio-Tek plate reader.

Serum measurements

Serum and whole blood samples were stored at -80°C prior to batch analysis. A serum cytokine multiplex array was performed by chemiluminescent immunoassay using an Evidence Investigator BioChip Analyser

Online supplements are not copyedited prior to posting.

© 2014 AMERICAN COLLEGE OF CHEST PHYSICIANS. Reproduction of this article is prohibited without written permission from the American College of Chest Physicians. See online for more details. **DOI:** 10.1378/chest.13-2483

(Randox Laboratories, UK) for the quantitative detection of interleukin-6, 8, 18 and monocyte chemotactic protein-1. Serum IGF-1, NT-pro-BNP and hs-CRP were measured by enzyme-linked immunosorbent assay (ELISA) (Siemens Healthcare Diagnostics, UK) and serum ACE-activity measured by a kinetic enzyme assay (Buhlmann Laboratories AG, Switzerland). A luminex based assay was used for fibrinogen measurement (Millipore, UK). For determination of ACE genotype, genomic DNA was extracted from whole blood and amplified by PCR using a 3-primer method with an I-specific oligonucleotide.

Intervention

Following quadriceps measurements, subjects were randomised to fosinopril (10mg) or placebo. Resting blood pressure and renal function were reviewed at one week by an independent assessor and if satisfactory the daily dose was increased to two capsules (fosinopril 20mg maximum). Dose was not escalated if systolic BP<110mmHg. Regular phone calls were made to the subjects by the independent assessor to ensure adherence with the trial medication (e-Table 1) and document any side effects. In those patients who completed the study, there were no reported issues with regards to trial medication adherence over the three month period. The reasons for dropout are shown in the consort diagram (Figure 1).

Online supplements are not copyedited prior to posting.

© 2014 AMERICAN COLLEGE OF CHEST PHYSICIANS. Reproduction of this article is prohibited without written permission from the American College of Chest Physicians. See online for more details. **DOI:** 10.1378/chest.13-2483

CHEST™ Online Supplement

e-Table 1: Trial schedule

	Day -7 Screening	Day 0 Treatment start[1]	Day 1	Day 7 Dose titration	Day 9	Day 28	Day 49	Day 90 End visit
History	X			X				X
Quadriceps function	X			X				X
FFMI	X							X
Bloods for genotype & inflammatory markers	X			X				X
U&E/FBC	X			X				X
SGRQ/CAT	X							X
Pulmonary function tests	X							X
Mid-thigh CT	X							X
ISWT	X							X
Quadriceps biopsy	X							X
Phone call			X	X	X	X	X	
Fosinopril (10-20mg) or placebo		X		X				

[1]Patients underwent pharmacy controlled (1:1) block randomisation. Due to expiry date of the trial medication, there was a pharmacy controlled batch change during the study, hence the final randomised allocation was 41 in the placebo arm and 39 in the treatment arm.

Online supplements are not copyedited prior to posting.

© 2014 AMERICAN COLLEGE OF CHEST PHYSICIANS. Reproduction of this article is prohibited without written permission from the American College of Chest Physicians. See online for more details. **DOI:** 10.1378/chest.13-2483

e-Table 2: Primer sequences (Real-time q-PCR)

	Forward sequence	Reverse sequence
**RPLPO**	5′-TCTACAACCCTGA AGTGCTTGATATC-3′	5′-GCAGACAGAC ACTGGCAACAT-3′
**Atrogin-1**	5′-GCAGCTGAACAA CATTCAGATCAC-3′	5′-CAGCCTCTGCA TGATGTTCAGT-3′
**MuRF-1**	5′-CCTGAGAGCC ATTGACTTTGG-3′	5′-CTTCCCTTCTGT GGACTCTTCC-3′
**IGF-1**	5′-CCACGATGC CTGTCTGAGG-3′	5′-TTTCAACAAG CCCACAGGGT-3′
**Myo D**	5′-GACGGCATGA TGGACTACAG-3′	5′AGGCAGTCTA GGCTCGACAC-3′
**TGF-β**	5′CCTGGCGAT ACCTCAGCAA-3′	5′CCGGTGACATC AAAGATAACCA-3′
**MHC I**	5′CCCTGGAGACTT TGTCTCATTAGG-3′	5′AGCTGATGAC CAACTTGCGC-3′
**MHC IIA**	5′TCACTTATGACTT TTGTGTGAACCT-3′	5′CAATCTACCTAA ATTCCGCAAGC-3′
**MHC IIX**	5′TGACCTGGGAC TCAGCAATG-3′	5′GGAGGAACAAT CCAACGTCAA-3′

Online supplements are not copyedited prior to posting.

© 2014 AMERICAN COLLEGE OF CHEST PHYSICIANS. Reproduction of this article is prohibited without written permission from the American College of Chest Physicians. See online for more details. **DOI:** 10.1378/chest.13-2483

RESULTS

e-Table 3: Baseline serum and vastus lateralis measurements

	Placebo group mean (SD)	Treatment group mean (SD)	p value
**Serum**			
ACE activity (IU/L)	48.0 (28.0)	43.5 (15.3)	0.43
HS-CRP (mg/L)	9.8 (21.0)	4.6 (4.6)	0.18
Fibrinogen (g/L)	2.6 (0.69)	2.8 (0.67)	0.20
IGF-1 (ug/L)	124.6 (61.5)	145.0 (103.8)	0.32
IL-6 (ng/L)	4.6 (15.2)	1.9 (2.5)	0.34
IL-8 (ng/L)	3.7 (3.5)	4.0 (2.8)	0.69
IL-18 (ng/L)	494.9 (332.7)	421.0 (174.7)	0.27
MCP-1 (ng/L)	66.2 (43.3)	77.7 (37.0)	0.26
**Vastus lateralis**			
Atrogin-1 mRNA (AU)	2.6 (0.5)	2.4 (0.5)	0.16
MuRF-1 mRNA (AU)	2.2 (0.7)	2.0 (0.8)	0.30
IGF-1 mRNA (AU)	0.9 (0.5)	1.0 (0.3)	0.56
Myo D mRNA (AU)	2.6 (0.6)	2.4 (0.7)	0.43
TGF-β mRNA (AU)	1.3 (0.7)	1.4 (0.6)	0.74
MHC I mRNA (AU)	3.9 (0.7)	4.2 (0.5)	0.11
MHC IIA mRNA (AU)	4.6 (0.5)	4.7 (0.6)	0.88
MHC IIX mRNA (AU)	4.0 (0.7)	4.1 (0.6)	0.78
4EBP-1 PHOS (AU)	2.2 (0.5)	2.0 (0.6)	0.56

Abbreviations: MuRF-1 – muscle RING finger protein-1; IGF-1 – insulin-like growth factor-1; ACE – angiotensin-converting enzyme; HS-CRP – high-sensitivity C-reactive protein; IL – interleukin; MCP-1 – monocyte chemotactic protein-1; TGF – transforming growth factor; MHC - myosin heavy chain; 4EBP-1 – eukaryotic initiation factor 4E-binding protein-1 .

Online supplements are not copyedited prior to posting.

© 2014 AMERICAN COLLEGE OF CHEST PHYSICIANS. Reproduction of this article is prohibited without written permission from the American College of Chest Physicians. See online for more details. **DOI:** 10.1378/chest.13-2483

e-Table 4: Serum and vastus lateralis measurements before and after 3 months of ACE-inhibition

	Placebo group (n=36)		Treatment group (n=31)		Change between groups
	Baseline	3 months	Baseline	3 months	p value
**Serum**					
HS-CRP (mg/L)	9.8 (3.5)	9.0 (4.3)	4.6 (0.8)	8.7 (2.3)	0.45
NT-proBNP (ng/L)	109.0 (16.6)	105.2 (16.5)	105.0 (11.5)	95.9 (11.8)	0.66
Fibrinogen (g/L)	2602 (115)	2771 (158)	2823 (123)	2873 (116)	0.49
IGF-1 (ug/L)	124.6 (10.3)	133.5 (9.7)	145.0 (18.6)	135.5 (12.7)	0.14
IL-6 (ng/L)	4.6 (2.5)	6.3 (4.1)	1.9 (0.5)	3.1 (1.5)	0.84
IL-8 (ng/L)	3.7 (0.6)	4.3 (0.9)	4.0 (0.5)	4.6 (0.7)	0.99
IL-18 (ng/L)	494.9 (55.5)	541.3 (65.0)	421.0 (31.4)	483.3 (50.0)	0.67
MCP-1 (ng/L)	66.2 (7.2)	63.7 (4.7)	77.7 (6.8)	71.8 (6.0)	0.63
**Vastus lateralis**‡					
Atrogin-1 mRNA (AU)	2.6 (0.1)	2.5 (0.1)	2.4 (0.1)	2.2 (0.1)	0.84
MuRF-1 mRNA (AU)	2.2 (0.1)	2.1 (0.1)	2.0 (0.2)	2.1 (0.2)	0.18
IGF-1 mRNA (AU)	0.9 (0.1)	1.2 (0.1)	1.0 (0.1)	1.4 (0.2)	0.65
Myo D mRNA (AU)	2.6 (0.1)	2.5 (0.1)	2.4 (0.1)	2.0 (0.2)	0.23
TGF-β mRNA (AU)	1.3 (0.1)	1.5 (0.1)	1.4 (0.2)	1.5 (0.2)	0.77
MHC I mRNA (AU)	3.9 (0.1)	3.7 (0.2)	4.2 (0.1)	3.6 (0.2)	0.06
MHC IIA mRNA (AU)	4.6 (0.1)	4.4 (0.2)	4.7 (0.1)	4.3 (0.2)	0.36
MHC IIX mRNA (AU)	4.0 (0.1)	4.1 (0.1)	4.1 (0.1)	3.9 (0.2)	0.64
4EBP-1 PHOS (AU)	2.2 (0.1)	2.0 (0.1)	2.0 (0.1)	2.2 (0.2)	0.08

Data are mean (SEM). ‡Vastus lateralis – placebo (n=28); treatment (n=22) due to insufficient biopsy sample for analysis.

Online supplements are not copyedited prior to posting.

© 2014 AMERICAN COLLEGE OF CHEST PHYSICIANS. Reproduction of this article is prohibited without written permission from the American College of Chest Physicians. See online for more details. **DOI:** 10.1378/chest.13-2483

e-Table 5: Baseline characteristics when stratified by ACE genotype

	DD (n=31)<br>Mean (SD)	ID (n=36)<br>Mean (SD)	II (n=13)<br>Mean (SD)	p value
Age	66.4 (8.3)	64.8 (7.7)	65 (6.9)	0.69
Gender	15/16	19/17	8/5	0.74
BMI (kg/m^2)	24.6 (5)	24.9 (5.2)	23.8 (4.6)	0.80
FFMI (kg/m^2)	17.4 (2.6)	17.2 (2.3)	16.6 (2)	0.63
FEV_1% pred	48.9 (18.8)	42.7 (22.7)	29.3 (10.6)	**0.01**
QMVC (kg)	25.7 (6.3)	25 (6.1)	23.2 (6.7)	0.49
$USRF_{CSA}$ (mm^2)	498 (127)	522 (123)	487 (133)	0.62

Online supplements are not copyedited prior to posting.

© 2014 AMERICAN COLLEGE OF CHEST PHYSICIANS. Reproduction of this article is prohibited without written permission from the American College of Chest Physicians. See online for more details. **DOI:** 10.1378/chest.13-2483

CHEST™ Online Supplement

e-Table 6: Change in quadriceps function and mRNA expression following 3 months ACE-inhibition when stratified by ACE genotype.

	Completion group (n=67) mean (SD)	Dropout group (n=13) mean (SD)	p value

	Treatment vs. placebo by ACE genotype (DD vs. ID vs. II) p value (ANOVA)	Treatment vs. placebo by ACE genotype (DD vs. ID and II) p value (ANOVA)
Endurance half-time (s)	0.68	0.74
QMVC (kg)	0.62	0.52
MT_{CSA} (cm^2)	0.43	0.73
Atrogin-1 mRNA	0.37	0.44
MuRF-1 mRNA	0.95	0.70
IGF-1 mRNA	0.99	0.96

Online supplements are not copyedited prior to posting.

© 2014 AMERICAN COLLEGE OF CHEST PHYSICIANS. Reproduction of this article is prohibited without written permission from the American College of Chest Physicians. See online for more details. **DOI:** 10.1378/chest.13-2483

e-Table 7: Completion group vs dropout group baseline characteristics

Age (years)	65.4 (7.6)	65.4 (9.1)	0.98
Gender (M/F)	37/30	5/8	0.27
BMI (kg/m^2)	25.0 (5.2)	22.9 (3.1)	0.17
FFMI (kg/m^2)	17.4 (2.4)	16.0 (1.8)	0.06
FEV_1% predicted	42.2 (20.7)	46.2 (20.4)	0.53
TLco% predicted	43.5 (21.0)	40.1 (14.5)	0.58
RV%TLC ratio	57.4 (10.5)	55.3 (9.2)	0.51
SGRQ (Total)	49.7 (18.8)	48.6 (20.8)	0.84
CAT score	21.8 (7.9)	22.3 (10.3)	0.83
Daily step count	4526 (3281)	4357 (3402)	0.88
ACE genotype (DD,ID,II) %	37/46/17	46/38/16	0.65
QMVC (kg)	25.6 (6.4)	21.7 (4.3)	**0.04**
TwQ (kg)	10.6 (3.4)	8.7 (1.2)	0.06
MT_{CSA} (cm^2)	94.7 (25.3)	84.9 (14.6)	0.18
ISWD (m)	241 (138)	229 (90)	0.78

(Dropouts were not included in the final per protocol analysis; this group were found to be weaker in QMVC, when compared to the completion group.)

Online supplements are not copyedited prior to posting.

© 2014 AMERICAN COLLEGE OF CHEST PHYSICIANS. Reproduction of this article is prohibited without written permission from the American College of Chest Physicians. See online for more details. **DOI:** 10.1378/chest.13-2483
